# Vitamin D_3_ Supplementation: Comparison of 1000 IU and 2000 IU Dose in Healthy Individuals

**DOI:** 10.3390/life13030808

**Published:** 2023-03-16

**Authors:** Eva Dědečková, Roman Viták, Michal Jirásko, Markéta Králová, Ondřej Topolčan, Ladislav Pecen, Tomáš Fürst, Pavel Brož, Radek Kučera

**Affiliations:** 1 Department of Pharmacology and Toxicology, Faculty of Medicine in Pilsen, Charles University, 323 00 Pilsen, Czech Republic; 2 Department of Immunochemistry, University Hospital and Faculty of Medicine in Pilsen, Charles University, 323 00 Pilsen, Czech Republic; 3 Faculty of Science, Palacky University in Olomouc, 779 00 Olomouc, Czech Republic; 4 Department of Clinical Biochemistry and Hematology, University Hospital and Faculty of Medicine in Pilsen, Charles University, 323 00 Pilsen, Czech Republic

**Keywords:** vitamin D, vitamin D deficiency, vitamin D insufficiency, supplementation, serum level

## Abstract

Background: Scientific studies point to a significant global vitamin D deficiency. The recommended dose of vitamin D for the adult population in Central Europe is 800–2000 IU/day. The aim of our study was to determine whether doses of 1000 IU or 2000 IU of vitamin D_3_ are adequate to achieve the sufficiency reference values of [25(OH)D]. Methods: Seventy-two healthy volunteers, average age twenty-two, took part in the study. The study was conducted from October to March in order to eliminate intra-dermal vitamin D production. Vitamin D_3_ in an oleaginous mixture was used. The participants used either 1000 IU or 2000 IU/daily for two 60-day periods with a 30-day break. Results: The dose of 1000 IU, taken for 60 days, increased vitamin D levels relatively little. Furthermore, serum vitamin D levels decreased in the 30 days following the cessation of supplementation. Taking 2000 IU daily led to a sharp increase in serum levels which plateaued 30 days after the subjects stopped using vitamin D_3_ drops. Conclusions: Both doses, taken daily, can help maintain adequate vitamin D levels during the winter months. A daily dose of 2000 IU, however, maintained the desired levels of vitamin D for a longer period.

## 1. Introduction

There has been a huge interest in vitamin D in recent years, not only in the fields of science and medicine but among the general public as well. This interest in vitamin D was sparked owing to a number of publications that have highlighted the worldwide vitamin D deficiency [1,2,3,4,5]. Vitamin D deficiency has been reported in 40% of the European population, 24% of the population in the USA and 37% of the Canadian population. Pregnant women, obese children and adults, populations with darker skin tone and people who rarely expose themselves to sunlight are all groups at risk of developing hypovitaminosis D [6,7].

Vitamin D belongs to the group of fat-soluble vitamins that are essential for human health. There are three ways for an individual to obtain vitamin D: exposure to ultraviolet light (UV), consuming vitamin D in food, or via supplementation [8].

We distinguish two basic forms of vitamin D: ergocalciferol and cholecalciferol. The chemical structures of ergocalciferol and cholecalciferol are not identical, but their metabolites show similar biological effects. Ergocalciferol, also known as a vitamin D_2_, is formed in the presence of UV radiation from its precursor, ergosterol. This occurs mainly in fungi, plants and yeast [9]. The dominant form in the animal world, and thus in humans, is cholecalciferol, or vitamin D_3_. Vitamin D_3_ is synthesized de novo in the skin from 7-dehydrocholesterol under the action of UV radiation. Depending on the wavelength, we divide UV radiation into UVA (315–400 nm), UVB (280–315 nm) and UVC (100–280 nm). UVB rays are responsible for the conversion of vitamin D_3_ from its precursors [10,11].

The axial tilt of the earth determines the amount of UVB that reaches the earth’s surface. In the winter months, when the angle of the sun on the horizon is lower than 45°, the rays take longer to go through the atmosphere and this results in an increased absorption of UVB radiation [12]. The amount of UVB radiation, and thus the amount of vitamin D_3_ that our bodies can produce, depends significantly on the latitude, season and time of day [13]. Only minimal or no vitamin D_3_ synthesis occurs at higher latitudes (above 33°) during the autumn and winter months and in the morning and late afternoon [14,15]. The synthesis of vitamin D_3_ is affected by a number of other external factors such as the weather, environmental pollution, layers of clothing and the use of sunscreen. Personal factors including genetic polymorphisms, age and skin colour also play a significant role since melanin in the epidermis significantly reduces the efficiency of UV radiation absorption [16]. 

Vitamin D_3_, having been synthesized in the skin, binds to a vitamin D-binding protein (VDBP) and is transported through the blood into the liver, where it undergoes hydroxylation and becomes hydroxyvitamin D_3_ [25(OH)D_3_], or calcidiol [17].

The biologically inactive form [25(OH)D_3_] is transported to the kidneys, where it undergoes further hydroxylation to its active form: 1α,25-d-hydroxyvitamin D_3_ [1,25(OH)2D_3_], or calcitriol [18]. The liver and kidneys are the main tissues responsible for vitamin D_3_ metabolism; however hydroxylases necessary for the synthesis of calcitriol are also present in various other tissues, including the skin.

Vitamin D itself has no significant biological activity. The effects of vitamin D are mediated through its active metabolite [1,25(OH)_2_D], which has the properties of a hormone and therefore vitamin D can be considered a prohormone [19]. The biological effect of [1,25(OH)_2_D] is manifested by the regulation of gene expression through binding to the vitamin D receptor (VDR) in the target tissue [20].

VDR can be found in almost every cell and tissue of the human body and this results in vitamin D having a very wide spectrum of effect [21]. The role of vitamin D in the regulation of calcium and phosphorus, a process that supports most metabolic neuromuscular transmission functions as well as the mineralization of bones, is widely acknowledged [22,23]. Vitamin D deficiency is associated with an increased risk of osteopenia, osteoporosis, fractures and rickets in childhood [24,25,26]. In recent years, there have been many studies that point to the extra-skeletal effects of vitamin D. Cell proliferation, differentiation and immune modulation are all affected by vitamin D levels [27,28]. Furthermore, vitamin D plays a role in a number of diseases [21,29], such as: type 1 and 2 diabetes [30,31,32], hypertension [33,34,35,36], cardiovascular disease [37,38,39], autoimmune diseases [40,41,42] and cancer [43,44,45,46].

Vitamin D levels are commonly determined based on a measurement of [25(OH)D], the main stock form of vitamin D. There is no global consensus on the [25(OH)D] concentration that defines vitamin D deficiency [6]. In Europe, a concentration of [25(OH)D] < 25 nmol/L has been considered critical for several decades now and a patient presenting with these levels is considered at risk of metabolic bone disease. According to the Endocrinology Society, blood serum levels of [25(OH)D] < 50 nmol/L are commonly considered a vitamin D deficiency, while concentrations of [25(OH)D] > 75 nmol/L are considered sufficient by most authors [15,47]. Various studies point to the fact that parathyroid hormone levels are only reduced in patients presenting with levels below 75 nmol/L of [25(OH)D] and thus concentrations of [25(OH)D] in a range of 75–125 nmol/L are commonly considered optimal for reducing the risk of developing secondary hyperparathyroidism and for maximizing musculoskeletal health [48,49,50,51]. Overall, values of [25(OH)D] up to 250 nmol/L are considered safe for children and adults [26].

Historically, vitamin D was mainly prescribed to prevent rickets. A dose of 200 IU/day of vitamin D_3_ was considered adequate for people under the age of fifty. However, in 2011, the US Institute of Medicine (IOM) established that a dose of 200 IU/day is insufficient. While this low dose addresses bone metabolism issues, it does not support the other important physiological functions of vitamin D [23]. The IOM then adjusted the daily vitamin D_3_ allowances to 400 IU for infants, 600 IU for children and 800 IU for adults; doses which are adequate for maintaining a concentration of 50 nmol/L of [25(OH)D] [52].

Nevertheless, a number of other studies have evaluated values of [25(OH)D] 50 nmol/L as insufficient [53,54,55,56]. The Endocrine Society in the USA recommends supplementing with a daily dose of 1000 IU for children who are over a year old and 1500–2000 IU for adults, so as to achieve a concentration of at least [25(OH)D] 75 nmol/L [48]. Daily vitamin D_3_ recommendations for Central Europe were published in 2013. The recommended dose for neonates and infants is 400–600 IU/day, for children and adolescents 600–1000 IU/day and for adults 800–2000 IU/day [57]. The aim of our study was to determine whether doses of 1000 IU or 2000 IU of vitamin D_3_ are sufficient for achieving and maintaining the reference values 75–200 nmol/L of [25(OH)D] in a sample of healthy young people during the winter months in the Czech Republic, a time when there is no natural synthesis of vitamin D.

## 2. Materials and Methods

### 2.1. Participants

A total of seventy-two healthy volunteers (fifty female and twenty-two male medical students), whose mean age was 22 years (20–27 years range). All participants filled in a questionnaire aimed at gathering information on the state of their health. Additional information about their type of diet, or use of hormonal contraception in women, was also collected as diet and hormonal contraceptives are known to influence changes in serum vitamin D levels.

The characteristics of the participants are summarized in Table 1. No additional supplementation of vitamin D in the form of medication or food supplements was allowed.

None of the participants was excluded based on the state of their health. No metabolic or endocrine diseases were reported.

This study was conducted in the period of October to March in order to eliminate the interference of intradermal production of vitamin D_3_.

### 2.2. Dosing Regimen

Participants were requested to take a vitamin D_3_ supplement according to the dosing regimen depicted in Table 2. An oral solution containing 0.5 mg/mL (20,000 IU) of vitamin D_3_ dissolved in an oleaginous mixture in a drip container was used. One drop of the solution is equivalent to 12.5 μg (500 IU) of vitamin D_3_ and the participants were instructed to use two to four drops daily for 60 days with a 30-day break after each 60-day period.

### 2.3. Blood Samples

The blood drawing schedule is depicted in Table 3. Blood was drawn five times in total over the course of six months.

Samples of peripheral blood were acquired using a VACUETTE^®^ CAT Serum Separator Clot Activator (Greiner Bio-One, Kremsmünster, Austria) serum collection tube. After cooling down the samples to room temperature, the tubes were centrifuged at 1700 rpm for 10 min in order to separate the serum from the whole blood sample. Every serum sample was then divided into three aliquots of an equal volume of 500 µL and stored at −80 °C before further analysis.

### 2.4. Sample Analysis

Measurement of total vitamin D levels was performed using the Access 25(OH) Vitamin D Total chemiluminescent assay in a Unicel^®^ DxI 800 (Beckman Coulter, Brea, CA, USA). This assay meets the Ghent University 25(OH) standard [58] and detects both 25(OH) vitamin D_2_ and 25(OH) vitamin D_3_. All samples were thawed at the same time and analyzed in one batch. All measurements were performed in accordance with the instructions provided by the manufacturers. 

Analyses of calcium, magnesium and phosphate concentrations were performed using the Cobas system (Cobas 8000 Analyzer, Cobas c702 module, Roche Diagnostics, Basel, Switzerland).

### 2.5. Statistical Analysis

Statistical analysis was performed using SAS, V. 9.4. (SAS Institute Inc., Cary, NC, USA) and MATLAB, V. R2007b (The Math-Works, Inc., Natick, MA, USA).

Discrete characteristics are expressed as frequency and percentages; continuous characteristics are expressed as mean, median, minimum, maximum, lower and upper quartile of serum vitamin D levels. The Chi-square test and Wilcoxon two-sample test were used to compare subgroups and determine statistical significance for categorical and continuous data, respectively.

Correlations were analyzed using Spearman rank correlation coefficient.

*p*-value < 0.05 was considered to be statistically significant.

Graphical statistical displays in the form of box–whisker plots and spaghetti plots are presented below.

For the power analysis, we assumed an increase in vitamin D from a mean of 75 nmol/L to a mean of 80 nmol/L after supplementation.

In accordance with previous studies, the standard deviation was assumed as 15 nmol/L. The correlation between the value before and after supplementation is relatively strong (r = 0.60 also according to previous experiences).

One-sided testing was assumed, and therefore one-sided Alpha = 0.025 (the same as two-sided Alpha = 0.0) and a power of 80% were assumed, due to the explanatory character of all the statistical analysis performed. 

The sample size estimation carried out prior to the study resulted in 71 subjects.

## 3. Results

The summarized results of measured vitamin D levels are shown in Table 4.

The mean initial concentration of serum [25(OH)D] was 75.50 nmol/L with a standard deviation of 20.30 nmol/L. The lowest recorded value was 28.18 nmol/L and the highest 134.47 nmol/L. One participant had deficient [25(OH)D] concentrations (28.18 nmol/L), six (8.3%) participants had insufficient [25(OH)D] concentrations (37.08–48.20 nmol/L), thirty-eight (51.4%) participants had [25(OH)D] levels lower than 75 nmol/L (50.21–74.58 nmol/L). Twenty-seven (37.5%) participants had adequate serum [25(OH)D] concentrations (higher than 75 nmol/L).

Following the 1000 IU vitamin D_3_ supplementation, the mean concentration rose to 83.43 nmol/L with a standard deviation of 20.77 nmol/L. The lowest recorded value was 51.89 nmol/L and the highest was 157.68 nmol/L. No participants had [25(OH)D] concentrations in the range of deficiency or insufficiency. Thirty (41.7%) participants had [25(OH)D] levels higher than 50 nmol/L but lower than 75 nmol/L (51.89–73.20 nmol/L). Forty-two (58.3%) participants had adequate [25(OH)D] concentrations ranging from 75.21 to 157 nmol/L. 

After the 30-day break in supplementation, the mean concentration fell to 66.54 nmol/L with a standard deviation of 15.70 nmol/L. One participant had deficient [25(OH)D] concentrations (34.25 nmol/L). Seven (9.7%) participants had insufficient concentrations (43.65–49.49 nmol/L). Concentrations lower than 75 nmol/L were found in forty-two (58.3%) participants (51.44–74.78 nmol/L). Twenty-two (30.5%) participants had adequate serum [25(OH)D] concentrations (76.63–117.15 nmol/L).

The mean concentrations of serum [25(OH)D] levels after 60 days of 2000 IU supplementation rose to 81.36 nmol/L with a standard deviation of 16.30 nmol/L. No participant had [25(OH)D] levels indicative of deficiency. One participant had insufficient [25(OH)D] levels (46.04 nmol/L). Twenty-seven (37.5%) participants had concentrations below 75 nmol/L (59.89 nmol/L–74.11 nmol/L). Forty-four (61.1%) participants had serum [25(OH)D] levels higher than 75 nmol/L (75.12–151.76 nmol/L).

Following the second 30-day break, the mean concentration of serum [25(OH)D] was 82.21 nmol/L with a standard deviation of 17.91 nmol/L. No participant had deficient [25(OH)D] concentrations. Two (2.8%) participants had insufficient [25(OH)D] concentrations (42.63–49.29 nmol/L). Twenty-four (33.3%) participants had concentrations higher than 50 nmol/L but lower than 75 nmol/L (54.21–74.46 nmol/L). Forty-six (63.9%) participants had sufficient [25(OH)D] levels (76.69–126.94 nmol/L).

**Figure 1:**
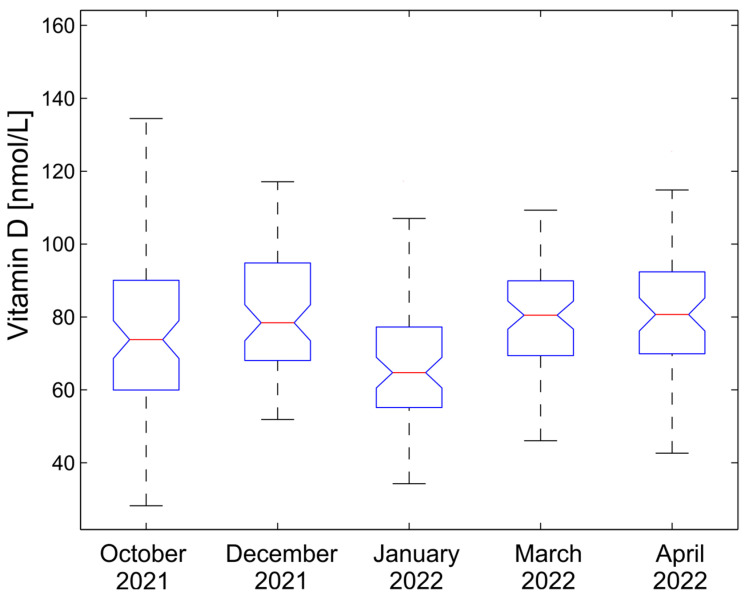
Distribution of vitamin D serum concentrations during the supplementation study.

A visual representation of the results is given in box plot Figure 1.

**Figure 2:**
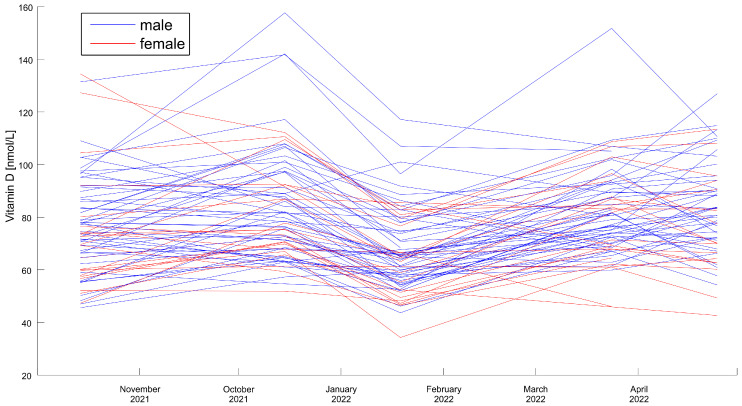
Visualization of individual changes in vitamin D concentrations in each participant.

The general trends in vitamin D concentrations in all individual participants are presented in a spaghetti plot in Figure 2.

We did not observe statistically significant differences between the sexes: the initial levels and the levels of vitamin D during the course of supplementation did not differ significantly. The results in men are slightly lower in each measurement, but without statistical significance.

**Figure 3:**
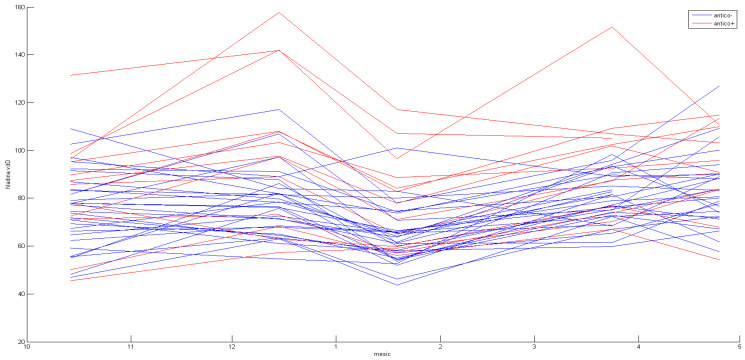
Distribution of vitamin D serum concentrations during the supplementation study: the effects of hormonal contraception.

[25(OH)D] concentrations were higher in female participants using hormonal contraceptives. There was a statistically significant difference in the increase between the first and second sampling (*p* < 0.0308), and then vitamin D levels stayed higher with varying statistical significance (*p* < 0.0308; *p* < 0.0242; *p* < 0.0501 non-significant, respectively). The results are presented visually in Figure 3. Detailed results can be seen in Table 5.

There was no statistically significant difference in [25(OH)D] concentrations in participants on vegan or vegetarian diets.

Mean concentration of serum calcium, magnesium and phosphates and their maximums and minimums are presented in Table 6.

## 4. Discussion

Vitamin D is an important micronutrient involved in a variety of physiological functions. There is, however, no consensus as to the vitamin D levels necessary for optimal health. The concentration of 50 nmol/L of [25(OH)D] is considered essential for maintaining adequate skeletal health [59,60], but no optimal concentration level has been established for extra-skeletal health. Concentrations of [25(OH)D] higher than 120 nmol/L have been shown to significantly decrease parathyroid hormone concentrations [61], significantly decrease adverse pregnancy outcomes [62] and enable the transfer of cholecalciferol into breast milk in breast-feeding women [63]. Concentrations of 120 nmol/L or above are common in individuals who are often exposed to sunlight: individuals who work outdoors and live in tropical and subtropical regions [64].

A group of participants, aged 22–27, was selected to describe changes in vitamin D levels in healthy subjects. A young cohort was chosen as older people are known to be more prone to vitamin D insufficiency, regardless of latitude [65]. Several changes in vitamin D metabolism are age-dependent. The absorption of calcium from the intestines decreases with age [66], the number of vitamin D receptors is lower in the elderly [67] and the hydroxylation of [25(OH)D] in the kidneys is reduced with aging as renal function decreases [68]. An oil-based oral solution was chosen for its assumed higher bioavailability in comparison to solid dosage forms [69].

The effectiveness of both supplementation dosage regimens was visible in our study. The study started with twenty-seven (37.5%) participants who had adequate serum [25(OH)D] concentrations (higher than 75 nmol/L). The first supplementation dose of 1000 IU, taken for 60 days, increased the mean vitamin D concentrations relatively little, with median values that ranged from 73.8 nmol/L to 78.4 nmol/L (6.2%). The change was, however, statistically significant, as *p* < 0.0288. Forty-two (58.3%) participants reached adequate [25(OH)D] concentrations ranging from 75.21 to 157 nmol/L. After the first dosage regimen, the participants took no supplements for 30 days, which explains the sharp drop in the median values from 78.4 nmol/L to 64.7 nmol/L; i.e., a drop of 13.7 nmol/L (17.5%) between the second and third blood sampling. This decrease was highly statistically significant: *p* < 0.0001. The second supplementation dose of 2000 IU, taken for 60 days, increased the mean vitamin D concentrations sharply. Between the third and fourth blood sampling the median increased sharply: from 64.7 nmol/L to 80.5 nmol/L; i.e., by 15.8 nmol/L (24.4%). This increase was highly statistically significant: *p* < 0.0001. Forty-four (61.1%) participants had serum [25(OH)D] levels higher than 75 nmol/L (75.12–151.76 nmol/L).

The value of vitamin D remained stable between the fourth and fifth sampling, with a negligible increase in the median from 81.4 nmol/L to 82.2 nmol/L; *p* = 0.5118. This stood in contrast to the first supplementation dose of 1000 IU, after which a significant decrease in [25(OH)D] concentration was observed during the first 30-day break from vitamin D_3_ drop administration.

While the daily dosing of 1000 IU cholecalciferol successfully increased the levels of serum vitamin D during winter months, a significant decrease in serum vitamin D was observed thirty days after discontinuation. A dose of 2000 IU increased vitamin D levels in a manner similar to the lower dose. There was, however, a minimal decrease in vitamin D levels 30 days after the supplementation ceased. Both 1000 IU and 2000 IU doses were therefore shown to be safe for substitution therapy. There were no cases of levels higher than 200 nmol/L, which is the upper limit of the laboratory reference values. Calcium and phosphate concentrations remained stable throughout this study.

There are very few other studies evaluating the effect of 1000 IU and 2000 IU vitamin D_3_ supplementation in a healthy population in the winter months at a similar latitude.

In 2017, Piltz et al. compared the effectiveness of vitamin D_3_ supplementation in a sample of young German women. Their study, which took place in the winter period and included two groups of patients, used multi-micronutrient supplements along with vitamin D3. In the first group of patients the daily dose of vitamin D_3_ was 200 IU, whereas in the second group the daily dose of vitamin D_3_ was 800 IU. In the 200 IU group, the required concentration of 75 nmol/L was reached in only 15.3% of patients, and in the 800 IU group, the required concentration of 75 nmol/L was reached in a mere 66.3% of patients [70].

In 2008, Holick et al. conducted a study on a sample of healthy adults in which they reported that even a daily dose of 1000 IU of vitamin D_3_ is not sufficient in order to achieve a concentration of 75 nmol/L [25(OH)D] in the blood during the winter months [71]. 

Diamond et al. published a 2012 study in which they provided vitamin D deficient patients with supplements. These patients were given a dose of either 2000 IU or 5000 IU of vitamin D_3_ for three months: from February to April. The study reported that only 43% of the patients taking the 2000 IU dose achieved the required concentration of 75 nmol/L, whereas 93% of the patients who were given 5000 IU reached the level of sufficiency. It is important to note that the patients were selected based on a pre-existing deficiency. The doses used in our study reflected a seasonal decrease in vitamin D in otherwise healthy individuals [72].

The available literature supports our observation that use of hormonal contraception proved to be associated with statistically significant higher [25(OH)D] plasma concentrations [73,74].

Vegetarians and vegans might be at risk of low vitamin D intake [75,76,77]. Our study, however, showed no statistically significant difference in [25(OH)D] levels between omnivores and vegetarians or vegans at any time point. Such results can be explained by the quality and adequacy of the participants’ vegan and vegetarian diet, since individual nutrient intake is known to vary significantly [78]. Furthermore, we had a relatively small sample size of vegans and vegetarians (8; 11.1% of all participants) and a majority of omnivores.

## 5. Conclusions

Our study is unique in its utilization of a group of healthy participants. The study thus puts forward the supplementation regimens that are suitable for a young healthy population during the winter months; a time when vitamin D production in the skin is low. The study is limited by its use of a relatively simple model of supplementation, without a cross-over or placebo control. It should, however, be noted that studies with a more complicated design are not common in vitamin D research.

Our results show that a daily dose of either 1000 IU or 2000 IU of vitamin D_3_ is an adequate choice for maintaining sufficient vitamin D levels during the winter months. A daily dose of 2000 IU was, however, observed to maintain the desired levels of serum vitamin D concentration for a longer period than a daily dose of 1000 IU and can thus be recommended for supplementing young healthy individuals during the winter months.

## Figures and Tables

**Table 1:** Characteristics of participants in this study.

Variables	Number	Percentage
Male	22	30.6
Female	50	69.4
All	72	100.0
Median of age	23 (20–27) years	
Hormonal contraception	14	19.4
Vegan/vegetarian	8	11.1

**Table 2:** Dosing regimen.

Month	Dose	Number of Days
October	1000 IU (2 drops)	60
November	1000 IU (2 drops)	
December	Break	30
January	2000 IU (4 drops)	60
February	2000 IU (4 drops)	
March	Break	30

**Table 3:** Blood draws.

No. of Blood Draws	Date	Relation to Vitamin D Supplementation
1	14 October 2021	Before the start of the supplementation
2	15 December 2021	After 60 days of 1000 IU supplementation
3	19 January 2022	After the first 30-day break
4	24 March 2022	After 60 days of 2000 IU supplementation
5	25 April 2022	After the second 30-day break

**Table 4:** Serum vitamin D concentrations before and after supplementation. All values are given in nmol/L.

Variable	Mean	Minimum	Maximum	Median	Lower Quartile	Upper Quartile
Before supplementation	75.5	28.2	134.5	73.8	60.0	90.1
After 60 days of 1000 IU supplementation	83.4	51.9	157.7	78.4	68.1	94.8
After the first 30-day break	66.5	34.3	117.2	64.7	55.2	77.2
After 60 days of 2000 IU supplementation	81.4	46.0	151.8	80.5	69.5	89.7
After the second 30-day break	82.2	42.6	126.9	80.7	69.9	92.4

**Table 5:** Serum vitamin D concentrations in female participants with and without hormonal contraception. Concentration values are given in nmol/L.

Hormonal Contraception	N	Sampling	Mean	Minimum	Maximum	Median	Lower Quartile	Upper Quartile
w/	14	1st	84.1	45.6	131.4	86.6	72.6	95.2
		2nd	100.5	57.3	157.7	97.5	73.1	107.9
		3rd	78.7	56.0	117.2	77.9	59.8	88.6
		4th	94.8	67.0	151.8	92.6	76.7	105.1
		5th	93.2	54.2	114.9	93.3	83.7	110.3
w/o	31	1st	74.7	47.0	109.1	74.6	62.3	83.6
		2nd	77.9	54.7	117.1	77.4	67.8	84.0
		3rd	64.1	43.7	101.0	62.6	55.0	70.8
		4th	78.9	59.9	98.3	77.2	72.5	85.0
		5th	83.1	57.7	126.9	80.3	72.4	89.9

**Table 6:** Serum concentrations of calcium, magnesium and phosphates. Values are presented as mean concentration with maximum and minimum given in brackets. All values are in mmol/L.

Variable	Calcium	Magnesium	Phosphates
Before supplementation	2.37 (2.11–2.60)	0.84 (0.75–1.06)	1.17 (0.67–1.54)
After 60 days of 1000 IU supplementation	2.34 (2.11–2.52)	0.83 (0.71–0.94)	1.11 (0.59–1.46)
After the first 30-day break	2.38 (2.16–2.58)	0.84 (0.70–0.99)	1.12 (0.68–1.53)
After 60 days of 2000 IU supplementation	2.37 (2.13–2.60)	0.84 (0.70–1.01)	1.08 (0.70–1.48)
After the second 30-day break	2.36 (2.14–2.55)	0.85 (0.73–0.98)	1.17 (0.73–1.68)

## Data Availability

Data are available from the corresponding author.
